# Supplementary material for: Dietary Patterns in Runners with Gastrointestinal Disorders
Source: Nutrients. 2021 Jan 29;13(2):448. doi: 10.3390/nu13020448 (PMC7912258; doi:10.3390/nu13020448)
Supplement: Supplementary file 1 [file nutrients-13-00448-s001.zip › nutrients-1082328 supplementary/FigureS1 Food Restriction in Running Questionnaire.pdf]

Food Restriction in Running Questionnaire

The purpose of this project is to learn about foods that endurance runners eat before they train or race, and if there are specific foods that they avoid. Gathered data will provide the sport science community with a better understanding of the most desirable pre-exercise nutrition message for these athletes. Furthermore, this research will determine the athletes' current attitudes toward food intolerances and training. The information obtained from these questionnaires will be kept strictly CONFIDENTIAL. Only members of the research team will have access to the answers. All data from the survey and documents are considered the property of the researcher. Any material written and/or published will be used in such a way as to not identify you. Your honesty and accuracy with this survey is greatly appreciated.

Please ensure that you have signed a consent form prior to completing this questionnaire. Please keep your copy of the consent form with your number in the event you wish to withdraw your information. Thank you.

Date:______________ Age:_____ Date of Birth:_______________ (yyyy/month/day) Gender: M / F

1. **What is the "performance" level that you usually compete in? Please check:**
   ☐ International/National ☐ Provincial
   ☐ Recreational (place top ½ of age group) ☐ Recreational (place lower ½ of age group) ☐ Do not compete

2. **On average, how many hours do you run each week? Please check:**
   ☐ 0 – 5 Hours ☐ 10 – 15 Hours ☐ 20 – 25 Hours
   ☐ 5 – 10 Hours ☐ 15 – 20 Hours ☐ More Than 25 Hours

3. **How many years have you included running as part of your training or exercise routine? Please check:**
   ☐ 0 – 3 years ☐ 3 – 5 years ☐ 5 – 7 years ☐ greater than 7 years

4. **What run distance do you compete in most often? Please choose one:**
   ☐ 5 km ☐ 6 - 10 km ☐ 11 - 20 km ☐ ½ marathon (21 km) ☐ 22 – 41 km ☐ marathon (42 km)
   ☐ Ultra-Distance (> 42 km) ☐ I don't compete in running races

5. **a) Have you ever been diagnosed with any of the following? Please check all that apply:**
   ☐ Celiac disease ☐ Inflammatory Bowel Disease (Crohn's disease or Ulcerative Colitis) ☐ Irritable Bowel Syndrome
   ☐ Heart burn/Reflux ☐ Hiatus Hernia ☐ Intestinal Parasites
   ☐ Other medical condition that restricts your diet? Describe: _____________________________________
   ☐ No Diagnosis for the above (proceed to Question #6)

   b) Who made this "diagnosis" as identified in Question #5a? Please check:
   ☐ Physician ☐ Naturopath ☐ Dietitian ☐ Holistic Nutritionist ☐ Chiropractor ☐ Allergist
   ☐ Self-Diagnosed ☐ Osteopath ☐ Other, Please List ____

6. **a) Are you <u>allergic</u> to any of the following foods? Please check all that apply:**

 Tree nuts Sesame Milk Soy Sulfites Whey Egg Whites
 Peanuts Fish/Seafood Wheat Gluten Mustard Casein Eggs
 MSG Other food allergies, please list: ______________________________
 No food allergies (proceed to Question #7)

b) Who diagnosed your <u>food allergy</u> as identified in Question #6a above? Please check:

 Physician Naturopath Dietitian Holistic Nutritionist Chiropractor Allergist
 Self-Diagnosed Osteopath Other, Please List ______________________________

c) What test was used to diagnose your food allergy as identified in Question #6b above? Please check:

 Unaware Skin prick test Blood test Urine test Feces test
 Hair test Saliva test Biopsy Other, Please List ______________________________

d) **If a blood test was used to diagnose your food allergy, what type of test was performed?**

 Unaware IgG IgE Other , please list ___________________

7. **Do you have any <u>food intolerances (foods that result in negative symptoms other than an allergy) not related specifically to running</u>? Please check all that apply:**

 Gluten free grain/cereal (e.g. rice, quinoa, pasta)
 Grain (e.g. granola bar, bread, pasta, bagel)
 Starchy vegetable (e.g. potato, sweet potato)
 Breakfast cereal, cold
 Hot cereal, (eg. oatmeal, Cream of Wheat)
 Yogurt
 Cheese
 Fruit _________________________
 Vegetables ___________________
 No food intolerances
 Legumes (e.g.lentils, chick peas, beans)
 Meat
 Poultry
 Fish/Seafood
 Nuts, nut butter
 Eggs
 Milk
 Lactose-free milk
 Other, please list: ______________________________
 Soy milk
 Almond milk
 Coconut milk
 Juice
 Coffee or tea
 Energy drink
 Sport drink
 Sport Bar or Gel

8. **a) When TRAINING are there any foods/fluids that you purposely AVOID in your pre-run MEAL or SNACK (0-4 hours before running TRAINING)? Please check all that apply.**

☐ Gluten free grain/cereal (e.g. rice, quinoa, pasta)
☐ Grain (e.g. granola bar, bread, pasta, bagels)
☐ Starchy vegetable (e.g. potato, sweet potato)
☐ Breakfast cereal, cold
☐ Hot cereal, eg. oatmeal, Cream of Wheat
☐ Milk products (milk, cheese, yogurt)
☐ Lactose-free milk
☐ Almond milk
☐ Coconut milk
☐ Soy milk

☐ Legumes (e.g.lentils, chick peas, beans)
☐ Meat
☐ Poultry
☐ Fish/Seafood
☐ Nuts, nut butter
☐ Eggs
☐ Fruit __________________
☐ Vegetables _________________
☐ High fibre foods in general

☐ Water
☐ Sport Bar or Gel
☐ Sport drink
☐ Juice
☐ Fruit/vegetable smoothie
☐ Coffee or tea
☐ Energy drink
☐ Chocolate

Other, please list: ____

☐ I don’t avoid any foods (proceed to Question #9)

b) When TRAINING what is/are your reason(s) for avoiding specific foods/fluids before running TRAINING? Check all that apply.

☐ Routine ☐ Previous bad experience ☐ Personal preference ☐ Received advice from someone
☐ Superstition ☐ Other, please explain ____

c) When TRAINING what symptoms might you experience during a TRAINING run if you had consumed a food/fluid that you typically avoid (as identified above) before training? Please check all that apply.

☐ stomach pain/cramps
☐ nausea/vomiting
☐ intestinal issues (pain, discomfort)
☐ reflux / heartburn
☐ no symptoms

☐ diarrhea
☐ bleeding
☐ urge to defecate
☐ bloating
☐ feeling of fullness or heaviness

☐ burping/belching
☐ side ache/stitch
☐ gas

☐ Other, please list: ____

9. **When TRAINING please describe what you would typically choose to eat and/or drink (including any supplements or special products) within the 4 hours before a TRAINING run:**

____
____
____
____

10. When RACING are there any types of food/fluid that you AVOID in your pre-race MEAL or SNACK (0-4 hours before running RACES/COMPETITIONS?)

a) Please check all that apply.

- Gluten free grain/cereal (e.g. rice, quinoa, pasta)
- Grain (e.g. granola bar, bread, pasta, bagel)
- Starchy vegetable (e.g. potato, sweet potato)
- Breakfast cereal, cold
- Hot cereal, (eg. oatmeal, Cream of Wheat)
- Milk products (milk, cheese, yogurt)
- Lactose-free milk
- Almond milk
- Coconut milk
- Soy milk
- Legumes (e.g.lentils, chick peas, beans)
- Meat
- Poultry
- Fish/Seafood
- Nuts, nut butter
- Eggs
- Fruit ________________
- Vegetables _______________
- High fibre foods in general
- Water
- Sport Bar or Gel
- Sport drink
- Juice
- Fruit/vegetable smoothie
- Coffee or tea
- Energy drink
- Chocolate

Other, please list: ____

 I don't avoid any foods (proceed to Question #11)

b) What is/are your reason(s) for avoiding specific foods/fluids before a running RACE/COMPETITION? Check all that apply.

- Routine
- Previous bad experience
- Personal preference
- Received advice from someone
- Superstition
- Other, please explain ____

c) When RACING what symptoms might you experience during a running RACE/COMPETITION if you had consumed a food that you typically avoid (identified above) before competing? Please check all that apply.

- stomach pain/cramps
- nausea/vomiting
- intestinal issues (pain, discomfort)
- reflux / heartburn
- no symptoms
- diarrhea
- bleeding
- urge to defecate
- bloating
- feeling of fullness or heaviness
- burping/belching
- side ache/stitch
- gas

 Other, please list: ______________________________________

11. When RACING Please describe what you would typically choose to eat and/or drink (including any supplements or special products) within the 4 hours before running RACES/COMPETITIONS:

____

____

____

____

12. Where do you receive information about nutrition for running? Rank up to your top 5 choices, "1" as most often and "5" as least often.

__ Internet (Blogs, Websites)	__ Pharmacist	__ Magazines
__ Social Media (Instagram, Twitter, Facebook)	__ Strength Trainer	__ Teammates
__ Naturopath/Chiropractor/Osteopath	__ Exercise Physiologist	__ Family / Friends
__ Coach	__ Physiotherapist/Athletic Therapist	__ Medical Physician
__ Dietitian	__ Holistic nutritionist	__ Health Food Store
__ I don't receive any information	__ Other athletes	

__ Other, please list ____

13. Have you ever attended a workshop(s) on nutrition for running?

☐ Yes ☐ No

14. How important is it to you to receive information about nutrition for running?

Very Important | Somewhat Important | Not Important

☐ 1 ☐ 2 ☐ 3 ☐ 4 ☐ 5

15. If you were to receive information about nutrition for running, how would you prefer to receive it? Please rank UP TO 3 responses with "1" as your "FIRST CHOICE":

__ Group presentations/workshops	__ Blogs or websites
__ Written material (hard copy, paper, magazine, book)	__ Social media (Instagram, Twitter, Facebook)
__ Individual consultation	__ Electronic newsletters

__ Other. Please List:_____________________________________

__ I do not want to receive information about nutrition for running

Thank you for taking the time to complete our questionnaire.
