# Supplementary material for: Dietary Patterns in Runners with Gastrointestinal Disorders
Source: Nutrients. 2021 Jan 29;13(2):448. doi: 10.3390/nu13020448 (PMC7912258; doi:10.3390/nu13020448)
Supplement: Supplementary file 1 [file nutrients-13-00448-s001.zip › nutrients-1082328 supplementary/Table S1 Customized Food Categories.pdf]

Table S1: Customized Food Categories

Table S1 Customized Food Categories provides example foods for the coding of the open-ended question "When RACING please describe what you would typically choose to eat and/or drink (including any supplements or special products) within the 4 hours before running RACES/COMPETITIONS".

Food Category	Example Foods
Gluten free grains	• Rice (brown, white, wild)<br>• Rice Cakes<br>• Gluten-free Bread<br>• Gluten-free Pasta<br>• Gluten-free Crackers<br>• Gluten-free Pancakes/Gluten-free Waffles<br>• Gluten-free Corn Tortillas<br>• Amaranth<br>• Arrowroot<br>• Buckwheat<br>• Millet<br>• Quinoa<br>• Sago<br>• Sorghum<br>• Teff
Grains containing gluten	• Bread/Toast/Bun/Roll<br>• Pasta (white or whole wheat or whole grain)<br>• Muffin<br>• Bagel<br>• Tortilla/Wrap<br>• Pitas<br>• English Muffin<br>• Croissant<br>• Granola Bar<br>• Pancakes/Waffles/Crepes<br>• Spelt<br>• Bulgur<br>• Wheat Berries<br>• Barley<br>• Couscous
Snack food salty	• Potato chips<br>• Tortillas<br>• Popcorn<br>• Pretzels<br>• Crackers
Breakfast cereal cold	• Wheat cereals<br>• Bran cereals<br>• Oat cereals (unless specifies gluten free)<br>• Granola (unless specified gluten free)

	• Muesli (unless specifies gluten free)
Breakfast cereal cold gluten free	• Rice cereals<br>• Any other cereals specifically noted as gluten free
Breakfast cereal regular hot	• Any hot cereal containing gluten<br>• Oatmeal (unless specifies gluten free)<br>• Wheat cereal<br>• Whole grain cereal
Breakfast cereal hot, gluten free	• Gluten free oats<br>• Gluten free oatmeal<br>• Rice cereal<br>• Porridge specified gluten free
Low FODMAP fruit	• Avocado<br>• Blueberry<br>• Raspberry<br>• Grapes<br>• Honeydew Melon<br>• Kiwi<br>• Lemon<br>• Lime<br>• Orange<br>• Pineapple<br>• Papaya<br>• Pomegranate<br>• Raisins, dried cranberries, currants<br>• Strawberries
High FODMAP fruit	• Apple<br>• Apricot<br>• Banana (ripe)<br>• Blackberry<br>• Cherries<br>• Dates<br>• Figs<br>• Gogi berries, dried<br>• Grapefruit<br>• Lychee<br>• Mango<br>• Nectarine<br>• Peach<br>• Pear<br>• Persimmon<br>• Pineapple, dried<br>• Plums<br>• Prunes<br>• Watermelon<br>• Dried Fruit

	• Fruit bars
Low FODMAP Starchy Veg (contain > 22 g carbs in ½ cup serving)	• French Fries<br>• Potato (mashed, boiled, baked, nuggets, wedges)<br>• Corn (limited to ½ cob)<br>• Sweet Potato (limited to ½ cup)<br>• Hashbrowns
Low FODMAP Vegetables (non-starchy)	• Alfalfa<br>• Bamboo Shoots<br>• Bean Sprouts<br>• Beets<br>• Bok Choy<br>• Broccoli<br>• Brussels sprouts<br>• Cabbage<br>• Lettuce, green and red<br>• Carrot<br>• Celery<br>• Chicory leaves<br>• Chili and Bell Peppers<br>• Chives<br>• Choy Sum<br>• Collard Greens<br>• Cucumber<br>• Eggplant<br>• Endive<br>• Fennel<br>• Ginger<br>• Green Beans<br>• Kale<br>• Lettuce<br>• Spinach or Spinach Salad<br>• Green or Yellow Beans<br>• Tomato
High FODMAP Vegetables (non-starchy)	• Asparagus<br>• Cauliflower<br>• Garlic<br>• Green Peas<br>• Leeks<br>• Mushrooms<br>• Onions<br>• Shallots<br>• Sugar Snap Peas
Legumes	• Lentils<br>• Chickpeas<br>• Hummus<br>• Black Beans

	• Pinto Beans<br>• Kidney Beans
Red Meat	• Meat<br>• Beef<br>• Pork (Ham)<br>• Bison<br>• Game<br>• Lamb<br>• Other Meats
Poultry	• Chicken<br>• Turkey<br>• Duck
Eggs	• Eggs<br>• Egg Whites<br>• Omelet
Soy/Meat Alternatives	• Tofu<br>• “Veggie” burger<br>• “Veggie” dogs<br>• “Veggie” ground beef<br>• Tempeh
Cured Meats	• Beef Jerky<br>• Deli Meat<br>• Sausages<br>• Turkey Jerky<br>• Bacon
Fish/Seafood	• Salmon<br>• Tuna<br>• Cod<br>• Haddock<br>• Trout<br>• Arctic Char<br>• Other fish
Nut/Nut butter	• Peanuts<br>• Peanut Butter (regular/light/natural/crunchy/smooth)<br>• Almonds<br>• Almond butter<br>• Walnuts<br>• Pecans<br>• Cashews<br>• Pistachios<br>• Other Nuts/Nut Butter
Seeds	• Chia<br>• Flax / Flax seeds<br>• Hemp<br>• Other Seeds/Seed Butter

Milk	• "Milk"<br>• Skim milk<br>• 1%, 2%, 3.5% (whole or homo)<br>• Chocolate
Milk alternatives	• Soy Milk/Beverage<br>• Almond Milk/Beverage<br>• Coconut Milk/Beverage<br>• Rice Milk/Beverage<br>• Cashew Milk/Beverage
Yogurt	• Regular<br>• Greek<br>• Plain<br>• Flavoured
Lactose-free Dairy Products	• Lactose-free Milk<br>• Lactose-free Regular Yogurt<br>• Lactose-free Greek Yogurt<br>• Lactose-free Chocolate Milk<br>• Lactose-free Cheese
Cheese	• Cheddar Cheese<br>• Mozzarella Cheese<br>• Cottage Cheese<br>• Babybel®<br>• Cheese Strings<br>• Cream Cheese<br>• Feta<br>• Other Cheese<br>• Parmesan
Juice	• Fruit juice (e.g. apple juice, orange juice, mango juice, etc.)<br>• Vegetable juice<br>• Tomato Juice<br>• V8®
Coffee/Tea	• Coffee<br>• Decaf Coffee<br>• Black tea<br>• Green tea<br>• Herbal tea<br>• Decaf Tea
Energy Drink	• Red Bull®<br>• Monster Energy®
Sports drink (contains carbs & electrolytes)	• 'Sport Drink' (when brand not given)<br>• Gatorade®<br>• Powerade®<br>• eLoad®
Electrolytes	• Nuun®<br>• 'Salt' tablets (brand not given)

	• Powerade Zero®<br>• eLoad zone tablets®<br>• Ultima®<br>• G2 – Gatorade®
Sports/Carbohydrate Bar	• 'Sport Bar' when brand not given<br>• Clif Bar®<br>• Honey Stingers Waffle®<br>• PowerBar®<br>• Luna®<br>• Lara bar®<br>• Fruit Source Bar
Protein Bar	• 'Protein bar' no brand given<br>• Clif Builder Bar®<br>• Pure Protein®
Sports Gel/Gummies	• 'Sport gel' (when brand not given)<br>• Clif blocks®<br>• Clif chews®<br>• Clif shots®<br>• GU®<br>• "Jelly Beans"
Other Beverages	• Pop / Soda<br>• Iced Tea<br>• Lemonade<br>• Alcohol<br>• Mineral Water<br>• Vitaminized Water
Sweeteners	• Maple Syrup<br>• Honey<br>• Jam/Jelly<br>• Agave (syrup)<br>• Sugar (white, brown, cane etc.)
Fats/oils	• Butter<br>• Margarine<br>• Any Vegetable Oil (canola, olive, sunflower etc.)<br>• Mayonnaise<br>• Salad Dressing<br>• Sour Cream<br>• Whip cream<br>• Coconut oil
Sandwiches	• Submarine/Sub Sandwich<br>• Sandwich<br>• Wrap Sandwich
Pasta Dish/Pizza	• Pasta with Tomato Sauce<br>• Pasta with Meat Sauce<br>• Lasagna

	• Vegetarian Lasagna<br>• Macaroni and Cheese<br>• Pizza
Burritos/Tacos	• Burritos<br>• Tacos<br>• Fajitas
Soups, Stews, Chilis and Stir Fry	• Soup<br>• Chili<br>• Vegetarian Chili<br>• Mixed Stir Fry
Other Combination Foods/Mixed Dishes	• Any other combination food that isn't on the lists
Dessert/Sweets	• Cakes<br>• Pies<br>• Brownies<br>• Cookies<br>• Pudding<br>• Ice Cream<br>• Pastries<br>• Donuts<br>• Candy<br>• Nutella<br>• Other Desserts
